# Examining Coroners’ Recommendations for Health and Safety Management of Ageing Heavy Vehicle Drivers: A STAMP Analysis

**DOI:** 10.3390/ijerph192316112

**Published:** 2022-12-01

**Authors:** Angela Batson, Sharon Newnam, Sjaan Koppel

**Affiliations:** 1 Monash University Accident Research Centre, Monash University, 21 Alliance Lane, Melbourne, VIC 3800, Australia; 2 School of Psychology and Counselling, Queensland University of Technology, Brisbane City, QLD 4000, Australia

**Keywords:** heavy vehicle drivers, ageing drivers, occupational health and safety, STAMP control structure, Coroner’s recommendations, road trauma

## Abstract

Recommendations were analysed from coronial cases involving ageing heavy vehicle drivers (≥55 years) and mapped onto a Systems Theoretic Accident Model and Processes (STAMP) control structure to identify the controllers and control actions influential in the heavy vehicle industry with regard to health and safety. A National Coronial Information System (NCIS) database search revealed 38 coroners’ recommendations arising from 14 unique cases of ageing driver involvement. There were no ageing themes identified in the analysis of coroners’ findings and recommendations. An examination of the STAMP control structure identified that the highest concentration of recommendations was in the level of regulation, the second most senior level of control, although safety constraints were advised for all five levels of the system. In regard to identifying themes of control flaws in the recommendations, the study found that “unidentified hazards” were the most common type of safety failure in the analysis of cases of ageing drivers, concentrated at the regulatory level, which indicates that additional risk identification methods by upper levels of control are needed. Therefore, a recommendation arising from the current study is that additional controls in safety intervention are necessitated in the upper and middle levels of the road freight transportation system; in particular, formalising health and safety education for organisational managers, with a focus on identifying ageing issues, would fill a gap in the system for managing ageing heavy vehicle drivers. In conclusion, this study has found that improving the health and safety of ageing heavy vehicle drivers necessitates additional safety constraints with a focus on formalised safety education for organisational managers, in addition to a means to detect emerging and unforeseen hazards in the road freight transportation industry.

## 1. Introduction

### 1.1. The Hazardous Heavy Vehicle Industry

Heavy vehicle driving is the most dangerous occupation in Australia, in regard to both worker fatalities and serious worker compensation claims for injury [1]. Each year, approximately 500 heavy vehicle occupants in Australia are hospitalised as a result of a vehicle incident, with around 30% of these being classified with a high-threat-to-life injury [2]. Also concerning is that between the years 2003 and 2015, there were 535 work-related fatalities within the road freight transportation industry in Australia [3]. As well as being a serious workplace matter, crash incidents involving heavy vehicles are a significant public safety issue. In 2019, of the 188 people who died in a crash involving a heavy vehicle truck, 89 were light vehicle occupants, 54 were heavy vehicle occupants, 17 were pedestrians, 17 were motorcyclists, and 10 were pedal cyclists [4].

These statistics highlight the fact that heavy vehicle incidents are a serious concern not only for the road freight transportation industry, but also for the society in which heavy vehicles operate. As of 30 June 2020, a total of 649,215 rigid, articulated, and non-freight-carrying road trucks were registered in Australia [5]. At a micro-level, each day, approximately 4200 heavy vehicles travel in the Sydney–Melbourne (two largest cities in Australia) corridor, constituting 25% of all vehicles travelling that thoroughfare [6]. It is important to understand this context, given that increasing amounts of trucks on the road are associated with higher rates of vehicle fatalities [7], with one study finding that a one-unit change in the percentage of trucks on the road is associated with more than 1 times the likelihood of a crash [8]. These figures highlight that the road freight transportation industry is both prolific and entrenched in society and that heavy vehicle safety is important for all road users. Thus, ensuring the health, safety, and wellbeing of workers in the transportation industry is a key concern, not only considering employer obligation from an occupational health and safety perspective but also to ensure public safety. 

Truck driving is the seventh most populous occupation in Australia with 191,000 workers being employed in 2020 [9]. The average age of a truck driver in Australia is 48 years, which is higher than the median age of 39 for Australian workers [9], and one-quarter of truck drivers are aged over 55 years [10]. These figures are important to consider as it is critical that intervention is targeted to meet the needs of this population.

The current study aimed to explore the scope of intervention targeting workers in the transportation industry, with a specific focus on situations involving ageing drivers of road freight transportation heavy vehicles. Due to the influences of ageing on driving skills [11,12,13], it is important to design interventions within this industry that are specifically suited to the ageing driver. Safety managers in the road freight transportation industry have described their older workers as a valued cohort of their driving workforce) [14]. Although managers have previously cited several challenges to the older generation of heavy vehicle drivers, such as their resistance to new technology, their unhealthy lifestyle behaviours, and communication style conflicts, overall their positive characteristics far outweighed the more challenging aspects [14]. Older professional drivers are experienced, loyal, demonstrate respect, exhibit a higher tolerance for hard work, and where necessary, can effectively implement compensatory strategies [14,15,16].

### 1.2. The Heavy Vehicle Context

The task of road freight transportation has serious implications for the health, safety, and wellbeing of those working in that industry. The lifestyle of a career on the road results in an unhealthy work environment, which has been described as a “healthy food desert” with limited access to nutritional food, as well as limited opportunities for physical activity, and disrupted sleep cycles [17,18,19]. These lifestyle factors of heavy vehicle drivers are associated with the development of medical conditions such as diabetes; concerningly, it has been found that untreated diabetes is associated with increased crash risk [20]. Heavy vehicle drivers are also susceptible to risks to their wellbeing, with issues such as stress, depression, isolation, and loneliness commonly reported [18,21]. Heavy vehicle crash incidents have been found to be associated with factors such as emotional stability and workload expectations [22]. In particular, excessive strenuous workload expectations, such as loading and unloading cargo, can increase driver stress, and exacerbate fatigue ” [23].

Significant risks have been identified for ageing heavy vehicle drivers. The research literature classifies a worker as an “older worker” at around 50 to 55 years [24,25]. As workers get older, their ability to work becomes increasingly affected by occupational environments that are hazardous and stressful [26]. Workers over the age of 65 years are more likely to develop chronic health problems which they need to manage at work [27]. Although some researchers have found that ageing heavy vehicle drivers do not have more involvement in crash incidents compared to younger drivers [16,28], older workers have been reported to need a longer recovery time following workplace incidents [29]. This is primarily due to a reduction in body resilience to trauma [30], due to ageing factors such as a gradual loss of muscle fibres, a lower rate of muscle repair, and a deterioration in motor and sensory functions [31,32]. Additionally, fatigue resulting from long-distance driving is also more significant in older drivers [16]. These risk factors highlight the importance of prioritising efforts on developing and maintaining the health, safety, and wellbeing of ageing workers in the road freight transportation industry. 

### 1.3. OHS Management of Heavy Vehicle Drivers

Every worker has the right to a safe work environment. Organisations have a “duty of care” to ensure the work environment is free of hazards, and they must provide their workers with the necessary assistance and training to help manage any potential risks to health and safety [33]. These duties are supported through legislation in some countries. For example, the Chain of Responsibility legislation, within the Heavy Vehicle National Law, was introduced in Australia in 2018 to recognise that it is not solely the heavy vehicle driver who is responsible for road transportation accidents and breaches of safety; each actor in the system who exercises control, influence, or demand regarding a transportation task is also responsible for maintaining a safe work environment [34]. In Australia, the Chain of Responsibility laws have been closely aligned with laws in workplace health and safety [35]. There are many actors responsible for safety breaches in the road freight transportation system under the Chain of Responsibility legislation, including consignors, packers, loaders, drivers, operators, schedulers, receivers, and employers or managers. Other countries that have similar Chain of Responsibility legislation include New Zealand, Canada, and South Africa [34,36]. 

One responsibility of an employer under a “duty of care” legal obligation is intervention to support and monitor the health, safety, and wellbeing of their workforce [33]. The organisational health culture, as well as the commitment of leadership, have both been identified as key forces in the creation of a healthy workplace. In support, it has also been found that the most influential factors in creating a safety culture were the management style’s emphasis on safety, as well as behavioural monitoring [37]. In a review of safety management in the heavy vehicle transportation industry, it was found that several management and operational factors were associated with a lower risk of crash incidents, including management commitment, safety policies, return-to-work policies, safety training, incentives, and scheduling or journal planning [38]. This research suggests that decision making by organisational management does make an impact on the health and safety of the workforce.

Unfortunately for heavy vehicle drivers, much of their time at work is spent in isolation on the road, and as a result, they tend to self-manage their own health, safety, and wellbeing needs [14]. This context presents a managerial challenge and an opportunity to focus research to address a gap in existing safety management practice. Specifically, despite the necessity to provide a safe and healthy workplace, there is a lack of research and guidance for employers in managing the health, safety, and wellbeing of drivers. This is particularly the situation when combining ageing worker concerns with occupational health and safety issues [39,40]. A possible reason for this is that many organisations still hold the belief that ageing workers are slowly withdrawing from full-time work after 50 years of age [41] and that these workers are not interested in professional development training [42]. Further to this is that older drivers are less likely to inform management of any decline in functional ability due to fear of repercussions [28]. Regardless of the reasoning, there is a need for targeted intervention to optimise the health, safety, and wellbeing of ageing drivers in the road freight transportation industry.

As a first step in addressing this need, it is important to understand the scope of intervention targeting ageing heavy vehicle drivers to inform the review and revision of current control measures to ensure alignment with key risk factors throughout the entire road freight transportation system, as well as the need to develop new controls where gaps in the system are identified. Thus, the first aim of the study was to identify the current scope of knowledge regarding critical safety recommendations for the management of ageing heavy vehicle drivers. Specifically, this study examined recommendations from coronial cases involving heavy vehicle drivers aged 55 years and older who were involved in a workplace transportation fatality in Australia.

### 1.4. Australian Coronial Data

Coronial investigation in Australia “*…*allow[s] a coroner to make recommendations as part of their finding following an investigation into a death or fire” [43]. The goal of the recommendations is to improve public health and safety by identifying opportunities for injury prevention [44]. The National Coronial Information System (NCIS) was established in Australia in 2000 to provide accessible internet-based coronial data to death investigators and researchers [45]. Coroners’ findings are considered facts based upon the evidence, although they do not result in a legal consequence; rather, legal causality is handled by the court system [46].

A systematic review [47] found 106 studies published between 2000 and 2014 that featured NCIS data as the primary source. Another review which looked at the period 2011 to 2017 reported that more than 215 publications were produced with NCIS data [45]. Thus, coroners’ findings and recommendations provide a suitable source of data to inform evidence-based prevention strategies and insight into critical safety issues. To explain further, coroners’ records of fatal vehicle crash incidents have been incorporated into research to advance learning in the populations of older road users [48], as well as for heavy vehicle drivers [49,50,51]. These studies incorporated the coroners’ data for purposes of examining the nature and extent of vehicle incidents, and to obtain an understanding of the scope of factors contributing to a crash. Coroners’ findings and recommendations have also been included in a STAMP control structure of a case study of a single fatal incident in the construction industry [52]. Furthermore, additional research has used coroners’ data on multiple cases to establish the road freight transportation system as a sociotechnical system which provided insight into the current level of knowledge related to risk factors in the industry [51]. 

Thus, coroners’ recommendations provide expert advice and education for public health researchers and policy-makers on the strengths and weaknesses in a safety system, and as a result, can facilitate opportunities for reform. However, coroners’ data have yet to be used to understand the scope of knowledge related to prevention strategies targeted at ageing heavy vehicle drivers, who have been identified as a unique and important cohort of the workforce [14,53]. It is anticipated that identifying gaps in the safety system by analysing critical safety issues arising from coroners’ recommendations will thus address the first aim of this study. 

The second aim of this study was to use a sociotechnical system thinking approach to map the breadth of recommendations identified in coroners’ reports across all levels of the road freight transportation system; specifically, the controllers and recommended control actions will be mapped on a control structure. The Systems Theoretic Accident Model and Processes (STAMP) approach by Leveson [54] is ideal for analysing complex sociotechnical systems. STAMP is suitable to the current study as its sociotechnical model enables the inclusion of indirect influential factors in the system, such as leadership and other organisational aspects, to be considered, rather than simply analysing preceding events leading to an accident [55]. STAMP is a suitable method for analysis of loss events as it can create an overview of the entire sociotechnical control structure, and identify failures in control and feedback mechanisms at each level of the system [56,57]. According to the theory created by Leveson [54], accidents occur as the result of inadequate constraints on safety behaviours on each level of the workplace system; a “constraint” is an acceptable method of achieving a safe system [58]. A defining feature of this approach is that STAMP [59] goes beyond the failure-based concepts in traditional safety models [60] to include analysis of control flaws in system interactions [54,58]. 

Within the STAMP approach, a “hazard” is defined as “A system state or set of conditions that, together with a particular set of worst-case environmental conditions, will lead to an accident (loss)”; with “safety” being “Freedom from accidents (loss events)” [58]. The control structure of a system is illustrated in STAMP using hierarchical levels which enables the controllers (i.e., the decision-makers and actors), controls (i.e., the interventions and strategies), and feedback to be identified at the level of decision-making influence in the system [54,61]. The STAMP control structure illustrates how incidents can emerge based on a series of design flaws, errors in decision making, environmental issues, organisational influences, or a lack of adequate regulation [54,62]. 

Research with the STAMP approach has been conducted widely in many industries such as construction [52], the long-distance oil pipeline transportation industry [63], residential aged care [64], warehouse logistics [65], and railway transportation [66] to understand the actors who share responsibility for safety and how each system can be optimised for mitigating risk. More specifically, the STAMP approach has been used to map the safety constraints in the road environment [67,68,69]. Previous research has utilised STAMP to represent the controllers, controls, and feedback mechanisms in a state road transportation system and identified that responsibility for road safety was diverse and distributed throughout all levels of the hierarchy [69]. An important finding from previous research was that road trauma does present as a systems problem in support of the STAMP approach of Leveson [54,69]. However, despite evidence of system hierarchy, other research has reported that a limited number of contributory factors are identified at the upper levels of the sociotechnical systems under examination in the literature [51,70,71]. A consequence of this focus on lower-level factors means that research on intervention strategies for the most influential controllers in the system tends to be limited [28,70,71]. 

Given previous research has established that the road freight transportation industry is a sociotechnical system [51,71], STAMP is an ideal model to map the current level of knowledge of social influence and technical issues in prevention activities (i.e., supplementary controls) using coronial data. An advantage of the STAMP control structure is that it is adaptable to the industry and purpose under examination [54]. The current study will thus adapt the generic STAMP control structure by Leveson (2004) [54] to suit the road freight transportation system in Australia. To develop the levels of the road freight transportation system, STAMP research by Salmon et al. (2016) [69] and Accimap systems thinking research by Newnam et al. (2017) [51] were used as the foundation. Thus, the STAMP control structure for this study will feature five hierarchical levels of the road freight transportation system, which will then be used to map the identified coroners’ recommendations: Level 1: Government and Legislature; Level 2: Regulators and Government Agencies; Level 3: Strategic Management; Level 4: Local Management; and Level 5: Operating Controllers, Equipment and Environment (see also Table 1). 

The controllers (as identified in the coroners’ reports) will be presented at their corresponding hierarchical level within the system and will be represented by individuals, organisations, departments, associations, or law-making bodies that the coroners specifically address in their recommendations. According to the systems thinking theory of STAMP, the controllers represented in the control structure must have a safety goal and be able to manipulate components in the system [54]. A traditional STAMP control structure also incorporates elements of system feedback into its design [54]; however, the data obtained from the coronial database will not provide information to create these feedback channels. The only feedback channel will be the coroners’ findings and recommendations. The STAMP control structure will thus focus on two main elements: (i) the controllers addressed by coroners in their recommendations and (ii) the coroners’ recommendations which will form the control elements in the control structure. An additional analysis in the study, following mapping of the STAMP control structure, will involve classifying the recommendations by themes of control flaws [54]; this comprises the third aim of the study and will be explained below. 

The control flaw classification provides a further understanding of the processes that lead to safety incidents and can provide additional insight into inherent weaknesses in the control structure of the system, rather than solely identifying component flaws [54]. Each coroner recommendation will be assigned to the control flaw that best describes the scenario that led to a fatality [54]. The classification system is organised around three main types of safety control flaws: (i) inadequate enforcement of constraints; (ii) inadequate execution of control actions; and (iii) inadequate or missing feedback [54].

Due to the unpredictable interaction of system components in a complex sociotechnical environment [60,72], there is a need to evaluate the safety constraints throughout the system [54]. Component failures are a common investigation focus of traditional methods of accident analysis, where it is theorised that increasing the reliability of a component will improve safety [59]. However, the systems thinking approach proposes that perfectly functioning components can lead to a hazard due to an error during component interaction; this is commonly called a system control flaw [59]. For example, in a STAMP study of an accident resulting from an oil pipeline leakage, a pattern of overlapping responsibilities was identified whereby different levels of the system had similar responsibilities in safety procedures, only it was to the detriment of safety prevention [63]. Detecting patterns of control flaws in a system is one way to plan intervention strategies [54]. For example, identifying a series of communication flaws whilst executing control actions can inform strategies to improve information channels [54]. Classification of control flaws can be useful for purposes of accident analysis research, as well as for prevention strategies in industries [65]. Other previous studies of accident investigations have also complemented their STAMP control structures with this classification of control flaws [55,65,68,73].

### 1.5. Study Aims

The first aim of this study was to identify the current scope of knowledge in health and safety of ageing heavy vehicle drivers by identifying recommendations for safety improvement from coronial cases involving drivers aged 55 years and older who were involved in a workplace transportation incident in Australia. The coronial cases will be searched for themes of ageing concerns.

The second aim of this study was to incorporate the coroners’ recommendations into a STAMP control structure by mapping the controllers (i.e., the decision-makers or actors), and the advisory control actions for each level of the system. It is important to identify the correct controllers in the road freight transportation system as the Chain of Responsibility legislation in Australia states that each actor who exercises control in the system is responsible for safety breaches [35].

The third aim of this study was to classify the coroners’ recommendations into Leveson’s classification of control flaws leading to a hazard [54,58]; detecting patterns in hazards may inform intervention prevention strategies by improving decision making and allocation of resources [74,75]. 

## 2. Method

### 2.1. Ethics Approval

Ethics approval was granted by the primary ethics committee, The Department of Justice and Community Safety Human Research Ethics Committee (JHREC), on 7 May 2021 to access data from the NCIS (all Australian states and territories except Western Australia) with the reference number CF/21/5115. Following JHREC approval, Monash University registered the project on 10 May 2021. Ethics approval from the Western Australian Coronial Ethics Committee was granted on 18 June 2021 with the reference number EC 21/2021.

### 2.2. Data Source

The data for this study were obtained from the NCIS database which hosts coronial cases from the year 2000 onwards. The NCIS database contains coded and free text variables on cases prepared by all state Coroners Courts of Australia (or coronial divisions of the state Magistrates Court) and New Zealand. NCIS data available for use in this study included case details, occupation details, time location, mechanism/object of injury, cause of death, police summaries of circumstances, and coroners’ findings and recommendations.

### 2.3. Study Population and Setting

The eligible study population comprised NCIS closed cases featuring heavy vehicle drivers aged 55 years and older who were involved in a workplace transportation incident in Australia between the years 2000 and 2021. 

### 2.4. Inclusion and Exclusion Criteria

The inclusion criteria included the following: heavy vehicle (trucks are classified as heavy vehicles in Australia if they have a gross vehicle mass (GVM) of more than 4.5 tonnes when fully loaded [76]) driver (except for emergency services and the construction industry); age of 55 years and older; involved in a workplace transportation incident (i.e., involvement is regardless of whether the heavy vehicle driver was the fatality, was injured, or not injured); closed cases of deaths reported from 1 January 2000 to the present (i.e., November 2021); incident resulting in death determined by the coroner as “unintentional”; and a recommendation, warning, or preventative comment made by the coroner. Recommendations not applicable to the heavy vehicle transportation sector were also excluded.

### 2.5. Case Identification

**Figure 1:**
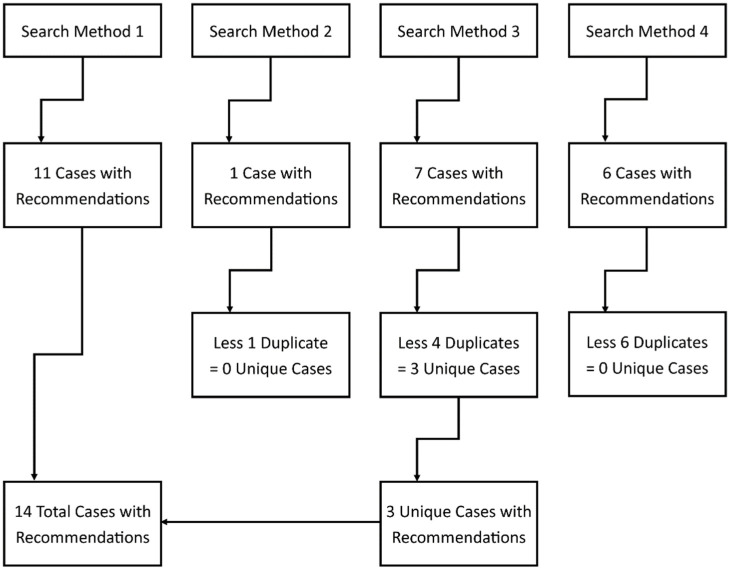
Case identification across the four search methods.

This study implemented four search methods to identify potential cases within the NCIS database. A diagram of the case identification process is presented in Figure 1.

(1) Search Method 1: The first variable was heavy vehicle drivers aged 55 years and older who died in a workplace transport incident. This search included the general term “driver” in the Occupation Text field, in addition to the label of “truck driver”, as this population may possibly be referred to as “Driver” or “Heavy Vehicle Driver” or similar. Other professional driver groups were then excluded. Additional search variables were as follows: case status (closed); work-relatedness; and case jurisdiction (Australia-wide). Case results were then examined to reveal if they had a “Yes” in the “Recommendation Made or Warnings Made” category.
(2) Search Method 2: Multiple-fatality incidents were also searched for potential cases. Cases were included if a recommendation or warning was made by a coroner, the case was closed, the case jurisdiction was Australia-wide, and the multiple-fatality incident was a “Vehicle Incident” described as “Land Vehicle—Multiple Vehicles”. This procedure was conducted in combination with various search terms including the following: (a) “truck*”; or (b) “heavy AND vehicle*”; or (c) “prime AND mover*”; or (d) “semi AND trailer”. Reports were then checked to determine whether the age of the heavy vehicle/truck driver was reported. If the age of the heavy vehicle driver was reported as 54 years or younger, the case was excluded. In addition, if the age of the heavy vehicle driver was not reported, the case was also excluded.
(3) Search Method 3: The NCIS releases regular reports on its website under the title of NCIS Fatal Facts which are summary reports of cases and the subsequent recommendations made by coroners throughout Australia. The Fatal Facts editions posted online feature cases from 2010 to the present (i.e., 2021 Edition 68). There is also an online feature with search options with the category tags “Transport and traffic related” and “Work related”. All available reports were hand searched for any relevant cases. Any potential cases were then checked to determine whether the age of the heavy vehicle driver was reported. If the age of the heavy vehicle driver was reported as 54 years or younger, then the case was excluded. In addition, if the age of the heavy vehicle driver was not reported, the case was also excluded.
(4) Search Method 4: An additional search method was used to identify any cases which may have been missed by the previous search methods. The search variables on the NCIS database in the Query Design screen included the following: the term “truck*” in a case report; the inclusion of “Recommendations Made or Warnings Made”; closed cases; case jurisdiction of Australia-wide; the “Blunt Force: Transport Injury Event” mechanism of injury; and finally, the object or substance producing injury being “Land Vehicle or Land Transport: Heavy Land Transport Vehicle”. If the deceased was not a heavy vehicle/truck driver, but a heavy vehicle driver was involved in the incident, the case was searched to check if the age of the heavy vehicle driver was reported. If the age of the heavy vehicle driver was reported as 54 years or younger, then the case was excluded. In addition, if the age of the heavy vehicle driver was not mentioned, the case was also excluded.

As shown in Figure 1, the initial NCIS database search identified 11 cases with coroners’ recommendations. The second search method identified one case, although this was a duplicate case. The third search method identified seven cases, although there were only three unique cases after the removal of duplicates. The fourth search method identified six cases which were all duplicates. Thus, in total, there were 14 unique cases which fit the inclusion criteria, and these featured 38 recommendations arising from the findings of coroners nationwide. 

### 2.6. Data Extraction and Analysis

A qualitative collective case study methodology was used to analyse the combined data [77,78,79], thus enabling a broader understanding of issues in the industry, as well as avoiding the breaching of case confidentiality. Data from the final list of 14 coronial cases were extracted into NVivo Qualitative Data Analysis Software program. In addition to the coroners’ reports (which included the recommendations), additional information collected included the following: age; sex; vehicle type; mechanism of injury; object causing injury; vehicles involved; area remoteness; interstate driver; time of incident; inquest held (yes/no); and work safety investigation conducted (yes/no). As per the requirement of using information from the NCIS, data were presented to minimise identification in cohorts of less than five cases. Thus, quantitative data on the aforementioned variables were converted to a qualitative format, rather than illustrated graphically or featured in a table. 

Qualitative data from case details, coroners’ reports and recommendations, and police summaries of circumstances were examined in the NVivo program, and coded into one of five hierarchical levels as described in Section 1.4. Coroners’ reports and police summaries of circumstances were also explored for potential themes of “ageing” and “older driver”. 

The first step in constructing the STAMP control structure was to map the recommendations to the applicable level of the system hierarchy; these recommendations served as the control actions of the STAMP control structure. The other fundamental component of the STAMP control structure involved identifying the controllers responsible for control actions at each level of the system. The controllers were the recipients of the recommendations, as identified in the coroners’ reports. For example, vehicle manufacturers were identified as a controller as a result of being specifically addressed in a coroner’s recommendation in regard to improving safety features on their vehicles and equipment. Other actors who were deemed to have a controlling influence in the system included law-making bodies, regulatory authorities, managers, and tactical decision-makers. 

Table 1 illustrates a comparison of the hierarchical levels of the road freight transportation system as shown in the current study, and as compared to the generic STAMP control structure by Leveson (2004) [54]. Table 2 includes examples of how the coroners’ recommendations were coded at each STAMP level.

In addition to being assigned to levels of hierarchy in the STAMP control structure, the identified recommendations were classified into the most appropriate type of control flaw as outlined by Leveson (2004) [54]. These themes of control flaws in a safety system will be used to identify patterns in the gaps of safety constraints throughout the system. Each recommendation was assigned to the most accountable level of control. For example, in the event that a new engineering safety standard is advised (but not mandated) for a vehicle, the recommendation will be assigned to the level of the manufacturer who has the decision-making power of whether to make the modification or decide against it.

**Figure 2:**
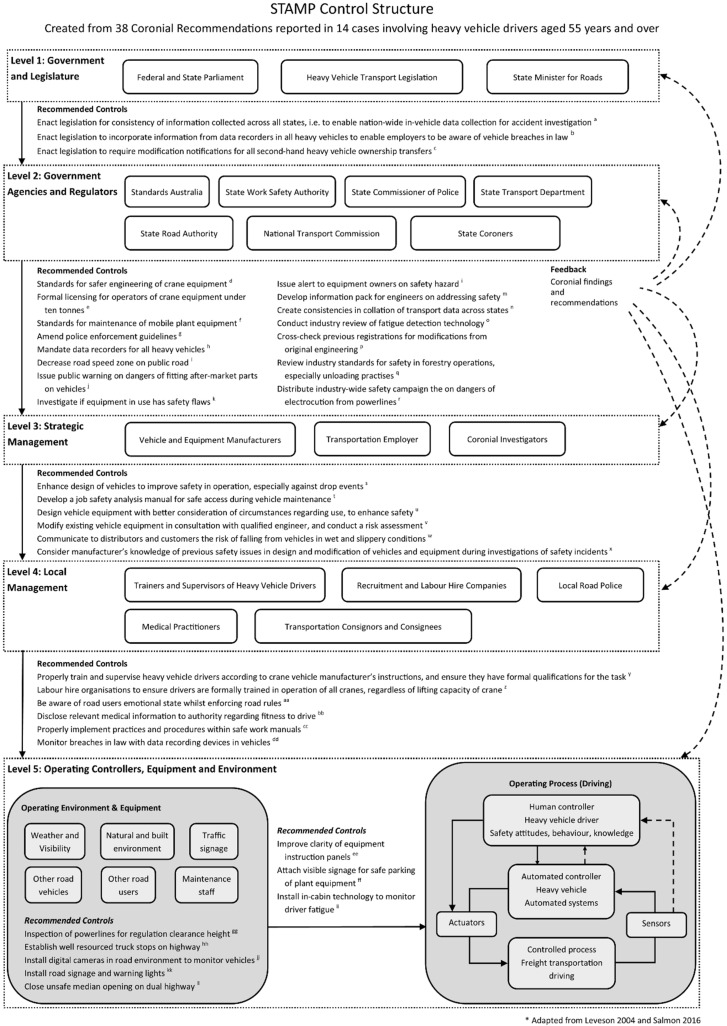
STAMP control structure of coroners’ recommendations for the road freight transportation industry (collective case study). * This figure is adapted from Leveson (2004) [54] and Salmon et al. (2016) [69].

In addition to being assigned to a hierarchical level in the STAMP control structure in Figure 2, control flaws leading to a safety hazard are also thus assigned to one of three broad categories: (1) inadequate enforcement of constraints; (2) inadequate execution of control actions; and (3) inadequate or missing feedback (Leveson, 2004).

## 3. Results

### 3.1. Overview of Cases

In the NCIS database, the current study identified relevant transportation incidents that occurred between 2002 and 2018. These transportation incidents included single-vehicle incidents (no crash involvement), single-vehicle crashes, and multiple-vehicle crashes. The most common mechanism of injury was a “transport injury event”, and the most common object causing injury was “land vehicle or land transport”. Coroners made recommendations most commonly in the Australian states of Victoria and Tasmania. 

The average age of the heavy vehicle drivers in the incidents was 62 years old (range = 55 to 60 s), all drivers were male, and the majority were driving a variation of a prime mover vehicle. The majority of events took place between 6.00 a.m. and 11.59 a.m., over a 24 h period of 6 h increments. In terms of the area remoteness classification, events occurred most commonly in outer regional and inner regional areas, followed by major cities. In the vast majority of the cases, the fatality occurred in the same state as the residential address of the driver. In most cases, there was no inquest held, and no official work investigation was conducted. 

There were no ageing themes or discussions of ageing or older drivers identified in the 14 included cases.

### 3.2. STAMP Control Structure 

The 38 recommendations identified from the 14 cases were mapped onto the STAMP control structure (see Figure 2) and assigned to one of the five levels. The first step in creating the STAMP control structure was to (i) map the controllers responsible for control actions at the most appropriate level of the system and then (ii) articulate the recommended controls in the system, based upon the coroner’s recommendations.

Identification of the controllers responsible for the control actions in the system was the first step in this approach. As explained previously, the assignment of the recommendation to a level was based upon the controller responsible for the recommendation. Furthermore, the most senior responsible actor for the advised control action was deemed as the “controller”; these controllers are represented in the STAMP control structure (see Figure 2) within a box at the appropriate level.

To explain, an engineering modification recommendation for manufacturers was assigned to Level 3: Strategic Management, as this level is responsible for decision making on manufacturing and product standards (as opposed to the tactical controllers who make day-to-day decisions on manufacturing at Level 5: Operating Controllers, Equipment, and Environment). The controllers in the system were represented by individuals, organisations, departments, associations, and/or law-making bodies to whom the coroners addressed their recommendations (see Table 3). For purposes of case confidentiality and structure generalisation, controllers such as government agencies and departments were allocated generic terms. All levels of the road freight transportation system were represented by multiple controllers in the system.

The mapping of the controllers thus determined where the recommended control action(s) would be placed in the hierarchy. For example, a state transport department responsible for amending traffic regulations is assigned to the level of the regulators and government authorities. Table 4 represents the number of coroners’ recommendations mapped at each level, and Table 5 presents a brief summary of coroners’ recommendations mapped at each level. 

### 3.3. Control Flaw Themes within the Road Freight Transportation System

In order to identify gaps in the existing control structure of the road freight transportation industry, the coroners’ recommendations were classified into categories of control flaws within the safety system as documented by Leveson (2004) [54]. Table 6, Table 7, Table 8, Table 9 and Table 10 feature the 38 recommendations alongside the assigned type of control flaw (Leveson, 2004) [54] at each level of the STAMP control structure, as classified by the current study. An explanation for the assignment of control flaws is presented within each table. As illustrated in Table 6, Table 7, Table 8, Table 9 and Table 10, “Inadequate Enforcement of Constraints: Unidentified hazards” (*n* = 13) was the most common classification of control flaws throughout the entire system, followed *by* “Inadequate Enforcement of Constraints: Inappropriate, ineffective, or missing control action for identified hazard: Design of control algorithm (process) does not enforce constraints: Flaw in creation process” (*n* = 7) and then “Inadequate or Missing Feedback: Not provided in system design” (*n* = 5). 

In regard to control flaws within the levels of system hierarchy, the most common assigned control flaw at the Regulator level was “Inadequate Enforcement of Constraints: Unidentified hazards” (*n* = 7/11).

Assigning the recommendations to one of the three broad categories of control flaws which can lead to a safety hazard identified that 63.2% of all recommendations were “Inadequate Enforcement of Constraints”, 23.7% of recommendations were “Inadequate or Missing feedback”, and 13.2% of recommendations were “Inadequate Execution of Control Action”. 

## 4. Discussion

This study aimed to identify the current scope of knowledge in intervention to improve the health and safety of ageing workers in the road freight transportation industry. This was achieved by examining 38 recommendations from 14 coronial cases with heavy vehicle drivers aged 55 years and older who were involved in a workplace transportation incident. This information was subsequently mapped onto a systems thinking STAMP control structure to assess the breadth of knowledge and responsibility for critical safety intervention and to discover reoccurring patterns of control flaws in the road freight transportation system.

The study found limited evidence of recommendations targeted at ageing heavy vehicle drivers, as evidenced by no specific mention of older or ageing drivers in reports by the coroners. A possible reason for this finding is that ageing heavy vehicle drivers may not be considered by coroners to be particularly unsafe drivers and thus to require recommendations for safer behaviour, especially given the system-wide stereotype of older workers being safer workers generally [80,81]. To further support this interpretation of the finding, previous research has identified that managers of ageing heavy vehicle drivers also consider ageing drivers to be safer drivers, as a result of their safety attitudes and work ethic [14], and display safer driving behaviours, even when compared to middle-aged drivers [28]. Another explanation could be that coroners are examining evidence one case at a time; thus, being isolated throughout different states, they may not be in the position to identify patterns and themes, which a collective analysis can achieve. STAMP theory advocates that feedback channels are important so each responsible actor can have an enhanced awareness of safety controls at the other levels of the system [58]. Thus, the collective coroners’ feedback in the current study contributes to knowledge in the system by allowing each individual coroner to have a broader view of issues within the system, and thus it creates an opportunity for more informed decision making. Regardless of these conjectures, given the ageing workforce and the importance of targeting intervention to align with the unique needs of this cohort, the current study has identified that there may be missed opportunities to address gaps in the current state of safety knowledge. 

This study mapped the recommendations from coroners’ reports onto a STAMP control structure [54] to illustrate the scope of knowledge from a systems thinking perspective. Controllers in the road freight transportation system, as indicated by the coroner in at least one recommendation, were identified across all five hierarchical levels. This finding was not surprising considering that Australia has a Chain of Responsibility legislation which identifies the roles and responsibilities of various controllers in the safety management of heavy vehicle drivers [35]. It is important that the responsible controllers are identified in the recommendations as untargeted recommendations are less effective for implementing preventative and protective measures [82]. However, it should be stated that some controllers in the road freight transportation system, as identified within the recommendations, are not included as actors in the Chain of Responsibility legislation, such as vehicle manufacturers who design and engineer safety features. A key reason for this is the Chain of Responsibility legislation is punitive, whilst coronial findings and recommendations aim to improve public safety [44,83]. Thus, the current study sought to contribute to knowledge on safety in the road freight transportation system, rather than to attribute blame to actors throughout the industry. 

Compared to the control structure by Salmon et al. (2016) [69], the current study was less detailed, particularly with regard to feedback channels; the only feedback in the current study was from the coroners themselves. A key similarity though is that both control structures mapped controllers of road safety at all levels of the system, thus providing evidence for shared responsibility for road safety, and the need for further identification of contributory factors at the upper levels of the road transportation system. 

Consistent with previous research [49,84], the current study found that the majority of coroners’ recommendations were directed at the level of regulators and government agencies with a suggestion of several control measures including the following: create and review standards; establish formal licensing; issue public safety alerts; and revise government department administration. This finding suggests that coroners view the role of regulators and government bodies to be as administrators of justice in safety management, as well as promoters of public safety messages [85]. Assigning the bulk of recommendations to regulators may be more in line with the coroners’ goal of generating preventative strategies for society. It could also be suggested that responsibility for safety reform in the system is thus assigned to independent governing bodies, rather than to the actors at the middle levels of the system, such as organisational managers, who have less of a societal focus.

Given this preference for assigning formalised recommendations to regulatory associations, a recommendation from the current study is that intervention could be advised in the form of regulations or standards for the safety education of organisational managers, especially in regard to ageing workers, where additional safety risks exist. It has been well acknowledged that there are challenges for employers and line supervisors in the management of work-related drivers [86,87], especially in addressing the needs of ageing heavy vehicle drivers [14]. Thus, additional intervention in the form of safety education and guidance in ageing issues could be formalised to mitigate these challenges. Given the limitations in investigation whereby coroners do not have adequate information around organisational activities to articulate specific recommendations targeted at each particular organisation [84], universal safety education targeted to road freight transportation industry managers would complement the public service objective of coroners’ recommendations. 

Providing additional support and guidance to organisational managers would work to fill the gap in recommended intervention between the higher and lower levels of the road freight transportation system as previously identified by Newnam et al. (2017) [51]. Research has found that middle levels comprising organisational managers have the greatest potential for control and responsibility within the system [64]. Additionally, it has been reported that the senior-level organisational management approach to safety issues plays a vital role in a workplace environment conducive to an accident occurring [88]. Thus, any successful system of workplace safety is dependent on better management of safety constraints by these actors in their managerial roles. 

Safety education, in a formalised structure, would benefit ageing heavy vehicle drivers consistently across the industry. Previous coronial research has supported recommendations for organisations in need of implementing change at the frontline level [82]. This recommendation of educating organisational safety managers would be in line with the two coroners’ recommendations identified in the current study, i.e., to conduct better supervision and training of drivers in the use of heavy vehicle equipment and to improve documentation of safe work procedures for workers in the lower levels of the system. A formalised structure would create a better alignment of safety knowledge across all organisations at all levels of the road freight transportation system.

Coroners’ recommendations are useful for creating awareness of unfamiliar issues within an industry [82]. The current study found that the majority of system control flaws were “unidentified hazards”, which provides evidence for gaps in the existing intervention for managing ageing heavy vehicle drivers. This finding suggests that the road freight transportation system is limited in its capability to optimise safety given the failure to previously recognise the potential for risk in the identified events. This gap in knowledge of safety hazards is particularly problematic for ageing heavy vehicle drivers, especially given that ageing itself can be considered a type of unidentified risk in the workplace due to the functional variability in how workers respond to hazard scenarios as they age in their work roles. As biological and cognitive functional changes are variable even between workers of the same age [40], individually monitoring ageing workers for potential health and safety issues is important. It has been reported that older drivers do not reliably self-regulate their driving behaviour [89]. Thus, workplace vigilance for unidentified hazards is especially important for ageing workers. The findings of this study suggest that governments and regulators need to empower employers with safety knowledge and hazard identification tools to better allow them to identify risks in ageing heavy vehicle drivers.

Several limitations were identified in this study. Firstly, some identified cases were not able to be included as the age of the heavy vehicle driver was not mentioned in case reports within the NCIS database. Often only the age of the deceased was mentioned in the case reports, and not the ages of all the involved parties. Access to more detailed descriptive information of persons involved in these cases could provide more data for analysis. A second limitation is that recommendations were more common in some Australian jurisdictions than in others, and thus, this analysis may not be indicative of the heavy vehicle industry nationwide. Thirdly, the coronial data did not provide sufficient information to understand and map the feedback channels in the system [54], which limited understanding of the processes used to facilitate communication and monitoring throughout the system. This information would be important to capture in future research to provide guidance on how regulators and government could best support employers in the safety management of ageing heavy vehicle drivers. Finally, this study was focused on Australian coronial investigations. Australia has a Chain of Responsibility which mandates the roles and responsibilities of particular actors in the system; thus, the findings of this study may not be transferrable to countries that do not have similar laws in place to regulate the responsibility of controllers in the safety management of heavy vehicle drivers. 

## 5. Conclusions

As a core concept of STAMP is a shared understanding and responsibility of the safety constraints throughout the system under examination, the current study has created additional knowledge into critical intervention needed to improve health and safety for ageing heavy vehicle drivers. Although no ageing themes were identified in the coroners’ findings, the collective case analysis of ageing drivers within the study provided some evidence of issues for this cohort. Although there were recommendations identified for improvement at all levels of the system, specifically, the analysis identified a preponderance of unidentified hazards at the level of regulation. The current study thus discovered that many safety hazards in the road freight transportation system may occur from a lack of adequate hazard detection, which then may contribute to situations leading to fatalities, particularly in cases of ageing heavy vehicle drivers. As the findings are specific to ageing drivers, hazard detection methods for this cohort should be enhanced throughout the system, particularly taking into consideration ageing issues affecting health and safety. The surfacing of unidentified hazards as a potential theme associated with the safety management of ageing heavy vehicle drivers suggests that there is a lack responsibility at the regulator level for risk identification for this cohort. As managers of heavy vehicle drivers have voiced a lack of upper-level guidance to address ageing risks in the workplace [14], it is suggested that standardised intervention be created by regulatory bodies for industry leaders and managers on how to detect and manage ageing issues for drivers in the road freight transportation system; this suggestion would help mitigate some lack of guidance for this cohort. Thus, ageing heavy vehicle drivers, who have the lowest capacity of control regarding health and safety in their hazardous driving role, can continue their work in a safer and more supportive environment. 

## Figures and Tables

**Table 1:** Comparison of System Hierarchy Levels.

Leveson 2004 Generic STAMP Levels	Study STAMP Levels
Level 1: Congress and Legislatures	Level 1: Government and Legislature
Level 2: Government Regulatory Agencies, Industry Associations, User Associations, Unions, Insurance Companies, Courts	Level 2: Regulators and Government Agencies
Level 3: Company Management	Level 3: Strategic Management
Level 4: Operations Management	Level 4: Local Management
Level 5: Operating Process	Level 5: Operating Controller, Equipment, and Environment

**Table 2:** Examples of Coroners’ Recommendations Coded at each STAMP Level.

Study STAMP Level	Example of Coded Coroners’ Recommendations
Level 1: Government and Legislature	“...review the relevant laws with a view to having Federal laws to protect the worker or consistent laws across the various States. This should include consistent provisions across the boarders for the retrieval of information from various agencies.”
Level 2: Regulators and Government Agencies	“My only observation at this time is, if it is “best practice” it should be formalised in a revised standard”
Level 3: Strategic Management	“However, the fact that a “drop event” can occur when the crane is incorrectly operated is a matter which, in my view, requires consideration by the manufacturer…”
Level 4: Local Management	“This is a timely opportunity to remind all medical practitioners of their obligation, not only to their patient but to the broader public safety, to disclose to the Registrar of Motor Vehicles relevant information relating to illness or injury likely to impact upon that patient’s ability to properly and safely drive a motor vehicle or other machinery where the public might be exposed to potential risk.”
Level 5: Operating Controller, Equipment, and Environment	“I will also request that consideration be given to improving the clarity of the instruction manual and the instructions displayed on the control panel…”

**Table 3:** Identified Controllers within the Road Freight Transportation System.

STAMP Level	Identified Controllers within the System
Level 1: Government and Legislature	Federal and state parliament, state minister for roads, and heavy vehicle transport legislation.
Level 2: Regulators and Government Agencies	National standards body, national transport commission, state commissioner of police, coroners, state transport department, state work safety authorities, and state road authorities.
Level 3: Strategic Management	Transport vehicle and equipment manufacturers, coronial investigators, and employer.
Level 4: Local Management	Trainers and supervisors of heavy vehicle drivers, recruitment and labour hire companies, local road police, medical practitioners, and transport equipment operators and contractors.
Level 5: Operating Controller, Equipment, and Environment	The operating controllers are the heavy vehicle driver (for example with levels of fatigue) and also the mechanics and maintenance staff. The equipment includes the heavy vehicles and the attached equipment. The operating environment includes other road vehicles, other road users, traffic signage, natural and built environment, weather, and visibility.

**Table 4:** Number of Recommendations Identified at each STAMP Level.

STAMP Level	No. of Recommendations
Level 1: Government and Legislature	3
Level 2: Regulators and Government Agencies	15
Level 3: Strategic Management	6
Level 4: Local Management	6
Level 5: Operating Controller, Equipment, and Environment	8

**Table 5:** Summary of Recommendations by Coroners at each STAMP Level.

STAMP Level	Summary of Coroners’ Recommendations at Each Level
Level 1: Government and Legislature	Enact safety legislation in parliament; create consistent laws across states
Level 2: Regulators and Government Agencies	Adopt standards in safety regulation; issue public safety alerts; conduct reviews of safety issues; necessitate formal licensing of training; refine government departmental administration; and amend traffic regulations
Level 3: Strategic Management	Enhance safety of vehicle and equipment via redesign and engineering; document and communicate safety issues; and re-evaluate accident investigation methods
Level 4: Local Management	Improve recruitment, training, and supervision of heavy vehicle drivers; disclose knowledge of safety issues; and implement safe work practices
Level 5: Operating Controller, Equipment, and Environment	Improve vehicle instructions and signage; install safety-related technology; conduct controller checks of the environment; and create safer facilities for drivers

**Table 6:** Coroners’ Recommendations Mapped at STAMP Level 1—Government and Legislature and Assigned to a Control Flaw (*n* = 3).

Coroners’ Recommendations	Control Flaw (Leveson 2004)	Explanation
(a) Law making bodies to review laws for worker protection, including the creation of consistent laws nationwide for retrieval of vehicle and accident information	Inadequate Enforcement of Constraints - Inappropriate, ineffective, or missing control action for identified hazard - Design of control algorithm (process) does not enforce constraints - Flaw in creation process	Decision-makers at the level of the state law have not collaborated with other state law decision-makers for consistency of laws for worker protection, leading to uncertainty in decision making, and a missed opportunity for clarity.
(b) Introduce legislation to incorporate information from data recorders in all heavy vehicles to enable employers to be aware of vehicle breaches in law	Inadequate or Missing Feedback - Not provided in system design	Advancements in technology have led to the invention of data recorders, although legislation has not kept up with novel technology, and this is a missed opportunity to capture law breaches which may compromise safety.
(c) State minister for roads to legislate notifications at point of sale to purchasers of second-hand heavy vehicles that the vehicle has been modified from its original engineering and design	Inadequate or Missing Feedback - Inadequate sensor operation (incorrect or no information provided)	There is an opportunity to capture safety-related data from decision-makers within government administration that would assist vehicle purchasers.

**Table 7:** Coroners’ Recommendations STAMP Mapped at Level 2—Regulators and Government Agencies and Assigned to a Control Flaw (*n* = 15).

Coroners’ Recommendations	Control Flaw (Leveson 2004)	Explanation
(d) National standards body to create a standard for safer design of cranes to ensure a “drop event” does not occur if the crane is operated other than in accordance with the manufacturer’s instructions	Inadequate Enforcement of Constraints - Unidentified hazards	No standard was created for the crane design to prevent a drop event, due to it not being identified as a hazard
(e) State work safety authority to require formal training and licensing by an independent and qualified person (not by employers) for all operators of vehicle crane equipment with lifting capacity under 10 tonnes (in addition to existing measures for above 10 tonnes lifting capacity)	Inadequate Enforcement of Constraints - Unidentified hazards	No formal training and licensing were necessitated for drivers as operating a crane with lifting capacity under 10 tonnes was not identified as a hazard.
(f) State government department to implement national standards for maintenance, service, and repair of mobile plant equipment	Inadequate Enforcement of Constraints - Unidentified hazards	There were no standards in place for the maintenance, service, and repair of equipment, due to these functions not being identified as a hazard.
(g) State commissioner of police to improve safety advice in the Motor Vehicle Stopping Techniques and Procedures document regarding a “corridor of safety” at a stopping location near traffic	Inadequate Enforcement of Constraints - Unidentified hazards	It was not adequately defined in police procedure that a corridor of safety was necessary near traffic in the event that a driver (being reprimanded) may get upset and move into the traffic zone, and as a result, be fatally injured.
(h) State transport department to mandate the fitting of data recorders to all heavy vehicles	Inadequate or Missing Feedback - Not provided in system design	Technology is now available to capture safety-related data in heavy vehicles. Fitting of this technology should be mandatory.
(i) State transport department to lower road speed zone and consider advisory speed signage designed specifically for heavy vehicles	Inadequate Enforcement of Constraints - Inappropriate, ineffective, or missing control actions for identified hazards - Design of control algorithm (process) does not enforce constraints - Process changes without appropriate change in control algorithm (asynchronous evolution)	A very busy section of road, with bends, needs a reduction in the speed zone to improve safety. Likely the volume of traffic flow has increased since the initial speed sign was installed.
(j) State road authority to issue public warning on the dangers of fitting after-market parts unless they have the same or equivalent properties of the part they are replacing	Inadequate Enforcement of Constraints - Unidentified hazards	There was no identification, at the regulatory level, that fitting non-genuine after-market parts to a vehicle was hazardous.
(k) State work safety authority to investigate if there are any vehicle spreader units in use that have a safety flaw whilst being operated in slippery and wet conditions, and if so, to advise the owners and contractors in writing of the need for modification	Inadequate Enforcement of Constraints - Unidentified hazards	There was no identification, at the regulatory level, that operation of the equipment during slippery and wet conditions was hazardous. There needs to be an investigation by regulators to find out if there are any other units in use.
(l) State work safety authority to issue an alert to owners and contractors about the risk of slipping and falling from machinery and trucks in wet and muddy conditions	Inadequate Enforcement of Constraints - Unidentified hazards	There was no identification, at the regulatory level, that operation of the equipment during slippery and wet conditions was hazardous. The regulator should issue an alert to owners and contractors.
(m) State work safety authority to develop an information pack for vehicle and equipment designers and engineers on how to address risk management and safety issues in developing and modifying equipment	Inadequate Enforcement of Constraints - Inappropriate, ineffective, or missing control actions for identified hazards - Design of control algorithm (process) does not enforce constraints - Flaw in creation process	The design process by engineers does not adequately address safety issues, and thus the regulator should develop an information pack on risk management for the engineers to follow.
(n) National transport commission to create provisions across all states in regard to retrieval of vehicle and accident information to protect all workers	Inadequate Enforcement of Constraints - Inappropriate, ineffective, or missing control actions for identified hazards - Process models inconsistent, incomplete, or incorrect (lack of linkup) - Flaw in creation process	Nationwide there are inconsistencies in state administration regarding collection of vehicle and accident information which would assist in injury prevention.
(o) An industry review should be conducted to identify what fatigue detection technology is available that would be suitable for integration with vehicle data recorders	Inadequate or Missing Feedback - Inadequate sensor operation (incorrect or no information provided)	An industry regulator should examine the feasibility of in-vehicle technology to provide additional feedback on driver fatigue.
(p) State road authority to cross-check previous registrations to enable identification of a vehicle that has been modified and that requires an engineering certificate (rather than relying solely on the accuracy of information provided in the transfer documents)	Inadequate or Missing Feedback - Not provided in system design	The second-hand vehicle registration process has no means of obtaining feedback on vehicle modification (beyond the transfer documents).
(q) State work safety authority to undertake a review of the Industry Standards/Safety in Forestry Operations, particularly in relation to loading and unloading of freight	Inadequate Enforcement of Constraints - Inappropriate, ineffective, or missing control actions for identified hazards - Design of control algorithm (process) does not enforce constraints - Flaw in creation process	The current standards in forestry operations do not adequately control safety in regard to loading and unloading practices.
(r) State work safety authority to distribute an industry-wide safety campaign on the danger of electrocution by contact with overhead powerlines on farm land	Inadequate Enforcement of Constraints - Inappropriate, ineffective, or missing control actions for identified hazards - Design of control algorithm (process) does not enforce constraints - Process change without appropriate change in control algorithm	Despite common knowledge that electrocution is hazardous, an additional control measure is needed to remind drivers to engage in safe work practices around powerlines on private farm land, in particular.

**Table 8:** Coroners’ Recommendations Mapped at STAMP Level 3—Strategic Management and Assigned to a Control Flaw (*n* = 6).

Coroners’ Recommendations	Control Flaw (Leveson 2004)	Explanation
(s) Manufacturer to enhance design of vehicle crane to improve safety in operation, such as to prevent a drop event if crane is operated other than in accordance with manufacturer’s instructions	Inadequate Enforcement of Constraints - Unidentified hazards	There was no indication at the manufacturing stage that the crane design would result in a hazardous drop event, if used contrary to manufacturer’s instructions.
(t) Employer to develop a Job Safety Analysis document for workers in the event that they need to conduct maintenance and/or cleaning of vehicle during operation	Inadequate Enforcement of Constraints - Unidentified hazards	There was no identification by employers that maintaining or cleaning the vehicle equipment during operation was hazardous, and thus necessitated a safe work procedure.
(u) Vehicle and equipment manufacturers to consider the circumstances under which their machinery will be used, and design the equipment to be more protective against obvious and known hazards, such as in slippery and wet conditions	Inadequate Enforcement of Constraints - Unidentified hazards	There was no identification at the manufacturing stage that maintaining or cleaning the vehicle equipment during operation was hazardous during slippery and wet conditions, and thus required additional safety engineering.
(v) Vehicle and equipment manufacturer to engineer modifications where handrails were not provided in the original design of the vehicle. These modifications would need consultation with a qualified engineer following a risk assessment and design brief.	Inadequate Enforcement of Constraints - Inappropriate, ineffective, or missing control action for identified hazard - Design of control algorithm (process) does not enforce constraints - Flaw in creation process	The initial design of the vehicle equipment did not have an appropriate handrail for safety purposes.
(w) Vehicle and equipment manufacturer to communicate to distributors and customers that there is a risk of falling whilst accessing vehicle in wet and slippery conditions	Inadequate Enforcement of Constraints - Unidentified hazards	There was no identification at the manufacturing stage that maintaining or cleaning the vehicle equipment during operation was hazardous during slippery and wet conditions, and following this discovery, communication should be issued to distributors and customers.
(x) Coronial investigators should consider the following whilst investigating cases: how safety issues are addressed in an original design brief; the manufacturer’s knowledge of previous safety incidents; any instruction or operators manuals; designer or manufacturer records; the number of units in the field; and how owners/operators are to be contacted in the event a safety problem is identified.	Inadequate Enforcement of Constraints - Inappropriate, ineffective, or missing control action for identified hazard - Design of control algorithm (process) does not enforce constraints - Flaw in creation process	The current investigation process by coronial investigators does not adequately cover relevant safety issues within the engineering process, especially in regard to manufacturer’s knowledge of past safety incidents.

**Table 9:** Coroners’ Recommendations Mapped at STAMP Level 4—Local Management and Assigned to a Control Flaw (*n* = 6).

Coroners’ Recommendations	Control Flaw (Leveson 2004)	Explanation
(y) Properly train and supervise heavy vehicle drivers according to vehicle manufacturer’s instructions, and ensure they have formal qualifications for the task	Inadequate Execution of Control Action - Communication flaw	There was a failure in the control commands whereby safety information was inadequately communicated to the driver by their supervisor.
(z) Labour hire organisations to ensure drivers are inducted and formally trained in operation of work equipment	Inadequate Execution of Control Action - Inadequate actuator operation	There was a failure in execution of a control action whereby an untrained driver was recruited to operate equipment on a vehicle, and although it was not anticipated that a safety incident would occur, the driver was not qualified to foresee or avert a hazardous situation.
(aa) Instructions for local police, whilst enforcing road rules, as to how to be aware of and then how to handle road users who may become upset	Inadequate Enforcement of Constraints - Unidentified hazards	There was no constraint in place to prevent the safety incident, due to there being no identification that the situation may become hazardous. Thus, no instructions were available to manage the situation.
(bb) Medical practitioners to disclose relevant fitness-to-drive information to Registrar of Motor Vehicles, regarding illness or injury, where the public may be exposed to potential risks	Inadequate or Missing Feedback - Communication flaw	There is a lack of feedback communicated to upper level controllers from medical practitioners regarding drivers’ illnesses and injuries relevant to fitness to drive.
(cc) Transportation consignors to properly implement the practices and procedures of their Safe Work Procedure Manual in relation to health and safety management	Inadequate Execution of Control Action - Inadequate actuator operation	The transportation consignor did not ensure that work practices were being conducted in alignment with their safe work procedure manual.
(dd) Managers to be made aware of breaches in law via the installation of data recording devices in heavy vehicles	Inadequate or Missing Feedback - Inadequate sensor operation (incorrect or no information provided)	Advances in technology have enabled an opportunity for managers to obtain feedback on driving law breaches with in-vehicle technology.

**Table 10:** Coroners’ Recommendations Mapped at STAMP Level 5—Operating Controller, Equipment, and Environment and Assigned to a Control Flaw (n = 8).

Coroners’ Recommendations	Control Flaw (Leveson 2004)	Explanation
(ee) Improve clarity of the crane instruction manual, as well as the instructions displayed on the control panel of the crane attachment on truck	Inadequate Execution of Control Action - Communication flaw	The instruction manual and the instruction panel do not adequately communicate safety instructions.
(ff) Attach a visible and accessible sign to forklift equipment warning operators not to park on an incline, or if unavoidable, to place blocks behind the wheels, in addition to not leaving these vehicles unattended	Inadequate Enforcement of Constraints - Inappropriate, ineffective, or missing control action for identified hazard - Design of control algorithm (process) does not enforce constraints - Flaw in creation process	Equipment within the work environment does not have appropriate signage for parking instructions, and thus is a safety hazard.
(gg) Conduct an inspection of powerlines to ensure they are installed at the prescribed clearance height	Inadequate Execution of Control Action - Inadequate actuator operation	There was no appropriate inspection of the height of the powerlines to assess proper clearance for safety purposes.
(hh) Establish well-resourced truck stops along a highway	Inadequate Enforcement of Constraints - Inappropriate, ineffective, or missing control action for identified hazard - Design of control algorithm (process) does not enforce constraints - Flaw in creation process	Highways which are frequented by heavy vehicles were not designed with appropriate rest stops for drivers.
(ii) Install technology in vehicle cabins to monitor heavy vehicle drivers for fatigue and to record information for purposes of investigation	Inadequate or Missing Feedback - Not provided in system design	Advances in technology have enabled an opportunity for fatigue monitors to be installed in vehicle cabins, thus enabling feedback on driver fatigue.
(jj) Install digital cameras in the road environment to monitor heavy vehicles across states	Inadequate or Missing Feedback - Not provided in system design	Advances in technology have enabled an opportunity for fatigue monitors to be installed in the road environment, thus enabling feedback on heavy vehicles, for safety purposes.
(kk) Installation of flashing amber lights at an advisory speed sign on a road frequented by heavy vehicles	Inadequate Enforcement of Constraints - Inappropriate, ineffective, or missing control action for identified hazard - Design of control algorithm does not enforce constraints - Process changes without appropriate change in control algorithm	An increase in traffic volume has created an unsafe road environment for the many heavy vehicles that travel that road. The installation of flashing amber lights would warn heavy vehicle drivers of the hazard.
(ll) Closure of a median opening on a section of road, in addition to the installation of warning signage prohibiting U-turns (or warning signage of dangers of encountering vehicles whilst performing U-turns)	Inadequate Enforcement of Constraints - Unidentified hazards	It was not identified that a median opening on a road is not a safe place to conduct a U-turn, and thus should be closed in the long-term, and have warning signage installed in the short-term.

## Data Availability

The data from this study was obtained from the National Coronial Information System database in Australia.
